# The Formation of an Interface and Its Energy Levels Inside a Band Gap in InAs/GaSb/AlSb/GaSb M-Structures

**DOI:** 10.3390/ma18050991

**Published:** 2025-02-24

**Authors:** Paweł Śliż, Dawid Jarosz, Marta Pasternak, Michał Marchewka

**Affiliations:** Center for Microelectronics and Nanotechnology, Institute of Materials Engineering, University of Rzeszów, Al. Rejtana 16c, 35-959 Rzeszów, Poland; djarosz@ur.edu.pl (D.J.); mruszala@ur.edu.pl (M.P.); mmarchewka@ur.edu.pl (M.M.)

**Keywords:** semiconductors, 2D materials, superlattices, infrared photodetectors, band gap, optical absorption spectroscopy, high resolution X-ray diffraction, k·p perturbation theory, Dirac fermions, computer simulations

## Abstract

We studied specially designed InAs/GaSb/AlSb/GaSb M-structures, a type-II superlattice (T2SL), that can serve as active materials for short-wavelength infrared (SWIR) applications. To obtain the dispersion relation of the investigated M-structures, k·p perturbation theory based on the eight-band model implemented in the nextnano++ v1.18.1 (nextnano GmbH, Munich, Germany) software was used. Numerical band-gap engineering and dispersion calculations for the investigated M-structures (composed of 6/1/5/1 monolayers, with InSb interfaces included) revealed the presence of an additional energy level within the energy gap. This energy level originates from the InSb-like interfaces and does not appear in structures with different layer or interface thicknesses. Its properties strongly depend on interface thickness, temperature, and strain. Numerical calculations of the probability density function ${\left|\Psi \right|}^{2}$, absorption coefficients, and optical absorption spectra at varying temperatures demonstrate that, under specific conditions, such as an optimised interface thickness and temperature, optical absorption increases significantly. These theoretical results are based on structures fabricated using molecular-beam epitaxy (MBE) technology. High-resolution X-ray diffraction (HRXRD) measurements confirm the high crystallographic quality of these M-structures.

## 1. Introduction

Experimentally proposed by B-M. Nguyen et al. in 2007 [1], the superlattice InAs/GaSb/AlSb/GaSb has been deeply investigated because its M-structure has a wide spectrum of valence band-tuning and engineering possibilities [2,3,4], allowing for an effective mass to be developed and ensuring its continued growth [1]. This experimental concept aligns with theoretical descriptions [5]. The M-structure can be used in two different ways: firstly, as a material for absorbers in the short-wavelength infrared (SWIR) range and, secondly, as a barrier material operating in mid-, long-, and very-long wavelength infrared (IR) radiation ranges. As the absorber, the M-structure can easily replace the binary type-II superlattices (T2SL) InAs/GaSb. It is essential to emphasize that, in theory, the InAs/GaSb superlattice is limited, and it is difficult to produce good-quality structure during the growth process. The proper binary InAs/GaSb superlattice should be about 2/8 ML [6] or 4/8 ML [7] (both cases pertain to the SWIR region). It has been proven that in situations when there is contact between the InAs and GaSb layers, for example, InSb or GaAs, the choice of a thin layer of InAs or GaSb does not compensate for the strain (compressive or tensile type). The M-structures that are usable in the SWIR range were described by, e.g., [4,8,9,10]. In the second example, the M-structure is usable as a barrier material for the holes in the T2SL InAs/GaSb superlattice photodetectors in the mid-wavelength infrared (MWIR) range [10] and long-wavelength infrared (LWIR) range [11].

In addition to their the application potential, the structures with a broken gap, such as the M-structures, have very interesting physical properties. Theoretical and experimental investigations show that in III-V quantum well (QW) [12,13,14], as well as in T2SL, e.g., InSb/InAsSb Gal-free T2SL [15,16], Dirac-like carrier dispersion can be observed. In the case of InAs/InAsSb T2SL structures, Dirac-like dispersion can be observed for structures with ultra-thin layers within the superlattice period from 3 to 6.2 nm, providing an effective band gap from 70 meV to 0 meV [15]. In Ref. [13], the authors define novel three-layer InAs/GaSb/AlSb QWs as having an “InAs geometry” or a “GaSb geometry” if they contain two symmetrical layers of InAs or GaSb, respectively. The “GaSb geometry” of such three layer InAs/GaSb structures is due to the presence of one InAs QW with two GaSb quantum barriers (QBs) and two symmetrical AlSb layers on both sides of the structures. Such architectures are similar to those of HgTe/HgCdTe or CdTe/HgCdTe QW, in which a Dirac-like dispersion can also be observed [17].

However, the results presented above, especially when we are talking about the three QW layers presented in Ref. [13], are theoretical predictions. When using real structures obtained via molecular-beam epitaxy (MBE) technology, it is necessary to take into account the interface layers that appear during the growth process. Such interfaces can be the result of non-intentional and intentional work. When we are talking about the use of M-structures for IR purposes (as a barrier material in absorber layers), the interfaces play a crucial role in obtaining the proper lattice constant that should match the GaSb buffer layers (or GaSb substrates). In such cases, InSb-like interfaces and/or GaAs-like interfaces should be created to ensure that lattice constant mismatches are minimal [18]. Such intentional interfaces influence the energy spectrum and effective energy gap, which is important for their application. Our first approach clearly shows that, besides the minimal lattice constant mismatch, which is a result of the use of proper interfaces, additional energy levels appear inside the energy gap. These additional energy levels are proved through calculating ${\left|\Psi \right|}^{2}$, which derives from the interface (IF) layers [18]. Many authors have presented numerical calculations for InAs/GaSb T2SL, taking into account the parameters of each compound, which are a part of such structures, e.g., [19]. In this work, we focused on M-structures, for which no such analyses have been conducted to date.

In this work, we focused on the properties of M-structures with additional energy levels. The numerical calculations were based on the real structures obtained by MBE technology. As we can see, this additional energy level has an energy dispersion relation that is characteristic of the Dirac-like dispersion and appears in special architectures with strictly defined InSb interface widths and well-defined temperature ranges. This case is strongly different from that presented for, e.g., T2SL InAs/InAsSb [15,16], as well as the three QW layers [13], and clearly shows that the interface layers should be taken into account in such cases. In the investigated M-structures, a Dirac-like dispersion relation is observed inside the energy gap, which is about 600 meV, which increases the maxima of the optical absorption at different temperatures.

## 2. Materials and Methods

All presented diagrams were created from data simulated using nextnano++ software (commercial Schrödinger–Poisson current solver) [20]. M-barriers were simulated as a 6-layer structure with an infinite number of periods and also as 5-period M-structures with 0.1 nm spacing in a one-dimensional grid. The order of layers in one period of the modeled M-structure was as follows (from value 0 nm on a horizontal scale, denoting the end surface of the entire M-structure, to the right of the *x*-axis in the substrate): GaSb/AlSb/GaSb/InSb/InAs/GaAs. The InSb (and GaAs) layer serves as the interface (IF) between the InAs layer and adjacent GaSb layers. The band edges, probability density functions, and dispersion diagrams were calculated using an 8-band k·p model (evaluated from 6-band k·p parameters) [21,22]. The k·p model used to calculate the dispersion diagrams included 16 energy levels of electrons and holes along the [010] direction that were additionally perpendicular to the surface direction ($k_{SL}$) in *k*-space for an infinite number of periods. To calculate the optical absorption spectrum for interband and intraband transitions, the quantum approach was used with the 8-band k·p model (evaluated from 6-band k·p parameters), with 80 electron and hole energy levels and dispersion relation calculations. The size of the $k_{\left\|(y,z\right)}$ space was set to ≤0.5 (2π/a), and a 33 × 33 point grid was used for spatial integration in absorption spectrum calculations (for circularly polarised light $\hat{\epsilon }=\hat{y}-i\hat{z}$, where *x* is the growth direction). The input files for absorption calculations were based on the “1D tutorial for interband transitions: Frankenberger” [23]. To reduce the computation time of the absorption spectra in nextnano++ for different parameter values, e.g., temperature, HTCondor was used as a functionality dedicated for distributed parallel computation in nextnanomat software [24], working on a cluster containing 15 compute nodes with a 4-core 3.2 GHz Intel i5-3470 processor and 16 GB RAM per node. The simulations included the evaluation of strain and lattice constants as functions of temperature. The material parameters used for simulations were taken from the material database included with the nextnano++ software.

The following parameters were used for the simulation:

(a) M-structure with an infinite number of periods:
  - The material layers were designed in a zinc blende structure.
  - The GaSb substrate’s orientation was set to x_hkl = [100] and y_hkl = [010].
  - The simulation grid size for the *x* growth direction was set to 0.1 nm.
  - For dispersion, with respect to the grid size of the |k| vector (for the direction perpendicular to the growth direction), it was set to about 0.05 nm^−1^, and for the $k_{SL}$ direction (along the growth direction), it was set to about 0.07 nm^−1^.
  - The temperature dependence of the energy gap and the temperature dependence of the lattice constant were turned on.
  - Calculated bands: Gamma, HH, LH, and SO.
  - Strain was set as pseudomorphic.
(b) Additional parameters were used for simulations for a 5-period M-structure (for dispersion relation and optical absorption):
  - 20 nm thick AlSb buffer (with 0.1 nm grid size).
  - 1 μm thick GaSb substrate (with 10 nm grid size).
(c) Additional parameters were used for simulations for a 5-period M-structure (parameters appropriate for optical absorption):
  - 0.5 size of the |k|-space (relative to the Brillouin zone) for the computation of optical transitions.
  - 16 |k| points (along the *y* and *z* axes) where optical transitions intensities were evaluated.
  - 8 points between |k| points where optical transitions intensities were interpolated.
  - Photon energy steps were set to 0.0001 eV.
  - The Gaussian broadening of energy levels for optical transitions was set to 1.0 × $10^{-6}$ eV.

## 3. Results and Discussion

### 3.1. HRXRD Measurements

The numerical calculations presented in this paper in Section 2 were carried out using the data (Table 1) obtained from the high-resolution X-ray diffraction (HRXRD) measurement of M-structures synthesized by MBE technology. Detailed information about the technological processes of investigated M-structures can be found in Ref. [18]. HRXRD measurements were performed using a Malvern Panalytical diffractometer with Cu K_*α*1_ radiation (λ = 1.540598 Å). The 2Θ−ω scan was recorded around the GaSb (004) reflection.

In the context of X-ray diffraction (XRD) analyses, the values of $Q_{x}$ and $Q_{z}$, which correspond to the components of the scattering vector in inverse space, proved to be crucial: $Q_{x}$ describes the component parallel to the sample surface, and $Q_{z}$ describes the perpendicular component. Analyses of the signal intensity distribution along these axes provided valuable information about the stress, composition, and thickness of the epitaxial layers. The coordinates of the diffraction space are expressed in terms of angular positions as follows:

$$Q_{x}=R\left(cos\;\omega -cos\left(2\theta -\omega \right)\right)$$

$$Q_{z}=R\left(sin\;\omega +sin\left(2\theta -\omega \right)\right),$$

where $R=\;|K_{H}|\;=\;|K_{O}|$, and the $K_{O}$—wave vector represents the incident beam, for which its length is $|K_{O}|\;=\;1/\lambda$. The $K_{H}$—wave vector represents a reflected beam that has the same length $|K_{H}|\;=1/\lambda$.

Analyses of the changes along $Q_{x}$ allows the assessment of stresses parallel to the surface and the identification of lattice mismatches in the epitaxial plane. However, the intensity distribution along $Q_{z}$ provides information about perpendicular stresses, layer thicknesses, and the ordering of the crystal structure. When an epitaxial layer grows on a substrate with a different lattice constant, stresses occur due to lattice mismatch. The shift of the peaks towards $Q_{x}$ indicates deformations in the plane of the layer (tension or compression). The shift of the peaks in the $Q_{z}$ direction is related to the perpendicular stresses resulting from the compensation of the lattice mismatch in the growth direction. Inverse space intensity mapping can reveal crystal mosaicism, i.e., the degree of rotation and displacement of crystal domains. Blurring in the $Q_{x}$ or $Q_{z}$ direction indicates the presence of defects such as dislocations that are the result of lattice mismatch. The width of the peaks along $Q_{x}$ and $Q_{z}$ reflects the density of dislocations and other defects. A high-quality crystal is characterised by sharp and well-defined diffraction peaks. Disorder (e.g., surface roughness or unevenness in the layer thickness) causes the peaks to be blurred.

**Figure 1:**
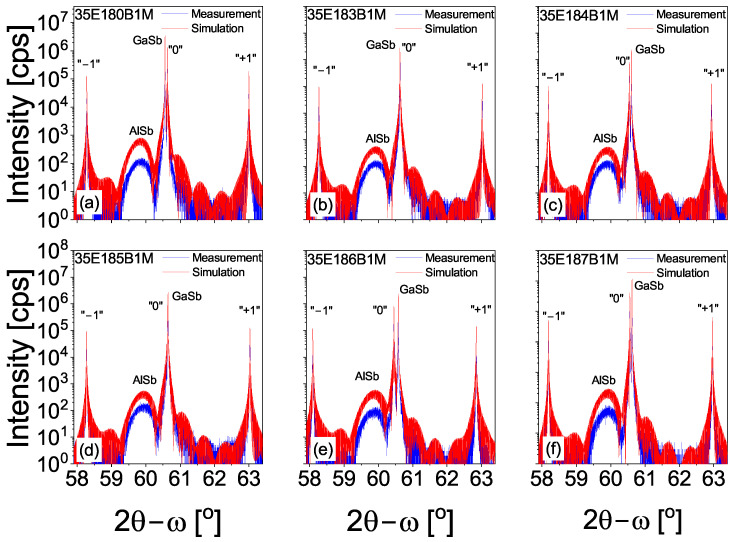
The 004 reflection 2Θ−ω scan and numerical simulation of the HRXRD measurements for the investigated M-structures. Markers: “−1” and “+1”—first order satellite peaks; “0”—zero-order satellite peak.

Each MBE process for each investigated sample was programmed to obtain different widths of InSb-IF, which were obtained using different Sb-soak times and different shuttering times of the In flux during IF growth. The results of the HRXRD measurements, together with the numerical simulations for all investigated samples in Figure 1, are presented. For all investigated samples, we were able to obtain a high and narrow peak, which were assigned to the GaSb substrate (see Figure 1), as well as the 0-th order peak, which originates from the T2SL M-structures. An additional peak from the 20 nm AlSb layers is also visible. Such thin films were grown between GaSb buffer layers and M-structures.

**Figure 2:**
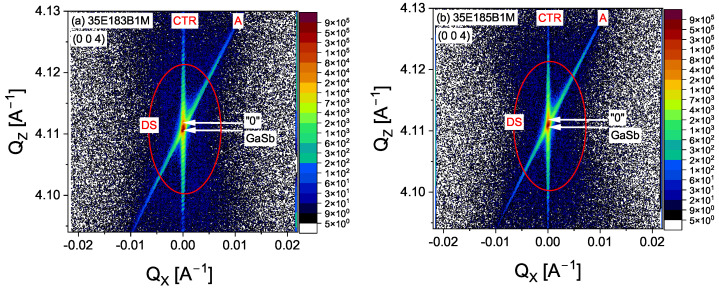
RSM maps for two investigated structures with the smallest measured lattice mismatch: (**a**) 35E183B1M and (**b**) 35E185B1M. In both panels, we marked the following: CTR—crystal truncation rod; DS—diffuse scattering (red ellipses); A—analyser streak parallel to the diffracted beam.

Figure 2 shows the reciprocal space maps (RSMs) for the two superlattices, which showed a smaller mismatch in the crystal lattice parameters. To make the results comparable, all structures were measured with the same parameters. The maps show that the reciprocal lattice points of the GaSb substrate and the ’0’ point are very close to each other, indicating minimal lattice mismatch. The lattice points of the zero-order satellite peaks are located in narrow ranges for the $Q_{x}$ and $Q_{z}$ axes, with values of −0.00040 Å^−1^ $\leq Q_{x}\leq$ 0.00071 Å^−1^, and −0.00028 Å^−1^ ≤ $Q_{z}$ ≤ 0.00059 Å^−1^. Both maps, (a) and (b), indicate an excellent fit of the structure to the substrate, with no evidence of relaxation in the overlying layer. The structures observed are of high crystal quality, as confirmed by the presence of a surface streak, which is known as a crystal truncation rod (CTR), on which Pendellösung peaks are visible along the $Q_{z}$ axis. Also noticeable on the maps are the streaks associated with the analyser, which run parallel to the diffraction beam (A). In addition, subtle diffuse scattering (DS) can be seen, and it is visible as red ellipses on the maps.

### 3.2. Nextnano++ Computer Simulations

**Figure 3:**
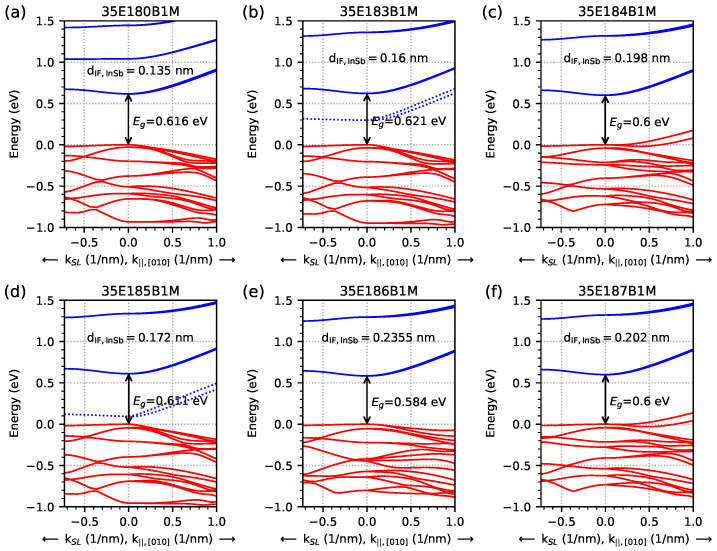
Dispersion relation, obtained at a temperature of 300 K from nextnano++ simulations, for the investigated samples (with an infinite number of periods) using data from Table 1. The red lines represent hole levels, and the blue lines represent electron levels. The dotted blue line represents the energy level that comes from the InSb interface and is visible only for the 35E183B1M and the 35E185B1M structures; it is manifested in this way at a temperature of 300 K. The presented values of the energy gap ($E_{g}$) were obtained at the Γ point.

The dispersion relation obtained from nextnano++ simulations for all investigated samples (with an infinite number of periods) using the data from Table 1 is shown in Figure 3. For values of *k* greater than zero, this concerns the dispersion relation for the *k*-vector parallel to the layers in the [010] direction. For negative values of *k*, the dispersion relation is for the *k*-vector perpendicular to the layer plane, and it is denoted by $k_{SL}$ (the 0 eV on the vertical energy scale is set to the energy value in the highest hole level at *k* = 0). The values shown for the energy gap ($E_{g}$) were obtained at the Γ point, calculated as the difference between the energy of the lowest electron level and the energy of the highest hole level (excluding the electron energy level coming from the InSb interface). The red/blue lines show hole/electron energy levels, and the blue dashed line shows the electron levels located inside the energy gap and those coming from the InSb interface (EGIF)—which occur at 300 K only for samples 35E183B1M and 35E183B1M, as shown in Figure 3b and Figure 3d, respectively.

**Figure 4:**
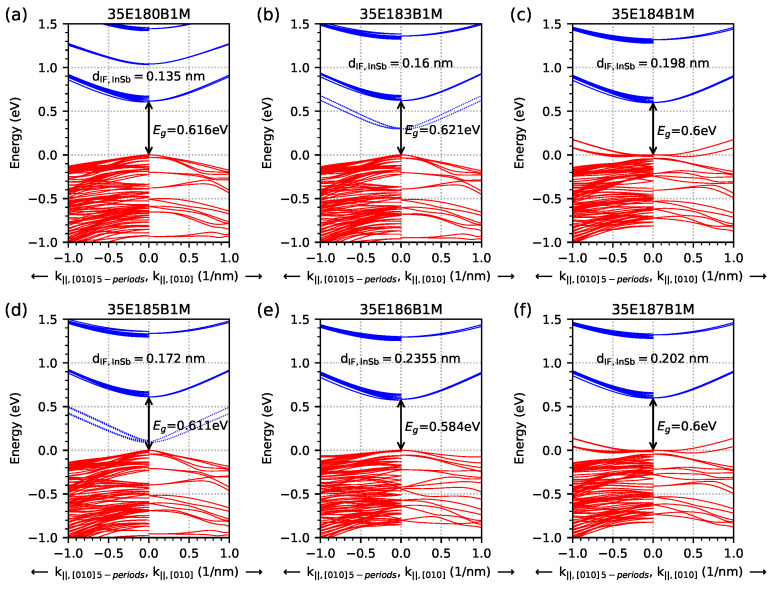
Dispersion relation obtained at a temperature of 300 K from nextnano++ simulations for the investigated samples modelled as a 5-period structure (as negative values of *k*) and an infinite number of period structures (as positive values of *k*), using data from Table 1 for the $k_{\left\|,[010\right]}$ direction. The red lines represent the hole levels, and the blue lines represent the electron levels. The dotted blue line represents the energy level coming from the InSb interface and is only visible for the 35E183B1M and 35E185B1M structures, and it manifests itself in this way at a temperature of 300 K. The presented values of the energy gap ($E_{g}$) were obtained at the Γ point.

For the calculation of the optical absorption spectrum, the samples were modelled as five-period M-structures with a 20 nm thick AlSb buffer and a 1 μm thick GaSb substrate (for quantum and optical calculations, the buffer and substrate are not included). For this reason, dispersion calculations were also performed for a five-period M-structures to compare with the results obtained for M-structures with an infinite number of periods, and the results are shown in Figure 4. On the left-hand side of each subfigure in Figure 4, there are results of calculations for the five-period model (with 80 electron and hole energy levels); on the right-hand side, there are results for the infinite number of period models (0 eV on the vertical energy scale is set to the energy value in the highest hole level at *k* = 0). It is worth noting that in the five-period model, the number of electron levels from the IF located inside the energy gap is equal to 10 compared to two levels for an infinite number of period models (this is visible as wider lines on the left side of the subfigures in Figure 4).

**Figure 5:**
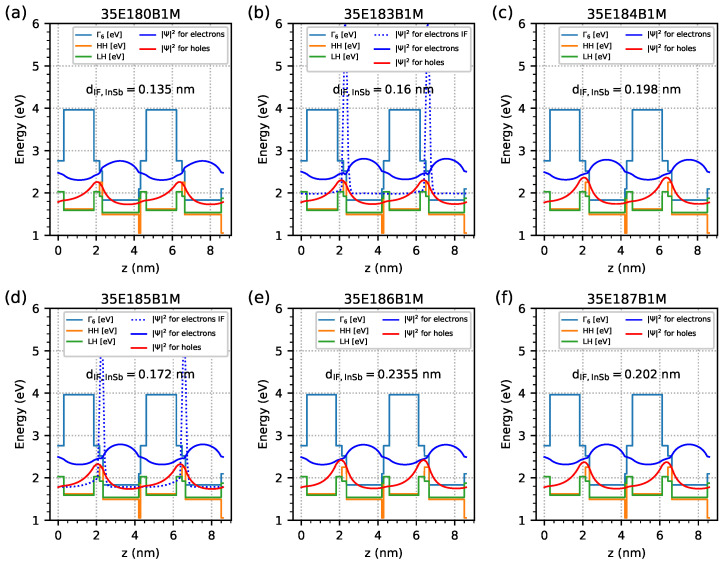
Band edge diagram and probability density function ${\left|\Psi \right|}^{2}$ for electrons and holes obtained at a temperature of 300 K from nextnano++ simulations with respect to the investigated samples modeled as an infinite number of period structures using data from Table 1. Band edge markers: $\Gamma_{6}$—electrons in the centre of the Brillouin zone; HH and LH—heavy and light holes, respectively. The red line represents holes, ${\left|\Psi \right|}^{2}$, and the blue line represents electrons, ${\left|\Psi \right|}^{2}$. The dotted blue line represents electron ${\left|\Psi \right|}^{2}$ coming from the InSb interface, and they are only visible for the 35E183B1M and 35E185B1M samples at a temperature of 300 K.

**Figure 6:**
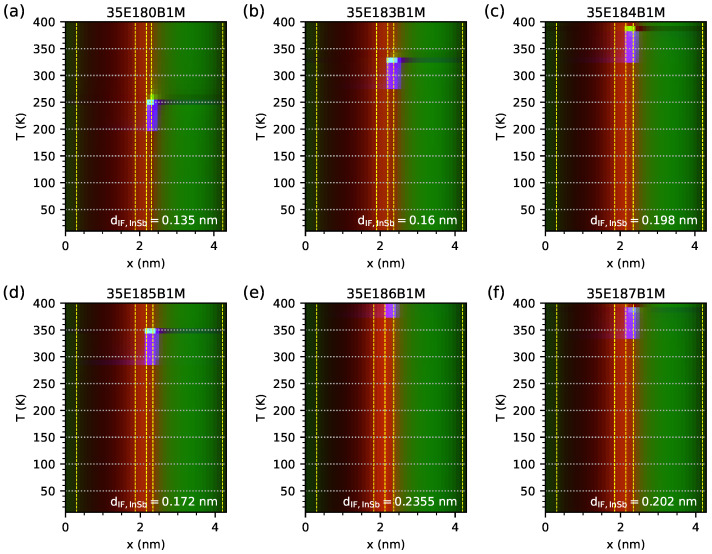
Probability density function ${\left|\Psi \right|}^{2}$ for electrons and holes obtained from nextnano++ simulations within the temperature range of 10–400 K for investigated samples modeled as an infinite number of period structures using data from Table 1. The red component of the colour represents the ${\left|\Psi \right|}^{2}$ corresponding to the highest energy level of the holes. The green component of the colour represents the ${\left|\Psi \right|}^{2}$ corresponding to the lowest energy level of electrons. The blue component of the colour represents the ${\left|\Psi \right|}^{2}$ corresponding to the energy level located inside the energy gap for the electrons from the InSb interface. The vertical dashed lines represent the boundaries of six materials within one period of the M-structure.

Figure 5 (corresponding to the results in Figure 3) shows the calculated band edge diagram and probability density functions (${\left|\Psi \right|}^{2}$) for electrons and holes obtained for a temperature of 300 K. The dotted blue line represents the ${\left|\Psi \right|}^{2}$ for EGIF and is only visible (at a temperature of 300 K) for the 35E183B1M and 35E185B1M samples. The ${\left|\Psi \right|}^{2}$ simulation results for the investigated samples and for a wide temperature range (from 10 K to 400 K) are shown in the form of a colour map in Figure 6, where the blue component of the colour represents ${\left|\Psi \right|}^{2}$, corresponding with EGIF. In this figure, it can be seen that the maximum value of ${\left|\Psi \right|}^{2}$ for the IF InSb region occurs within a limited specific temperature range, and it is visible in purple for lower temperatures (as a sum of red and blue colours) and in cyan (as a sum of green and blue colours) on the map.

**Figure 7:**
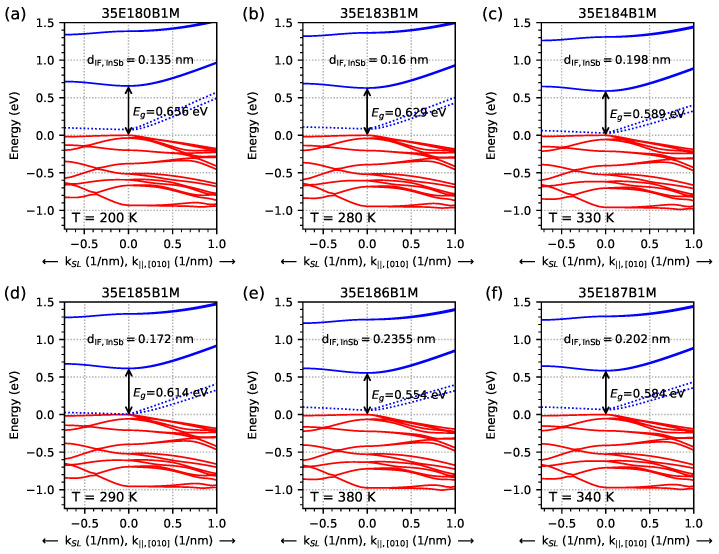
Dispersion relation for investigated samples parallel and perpendicular to the sample layers for the *k*-space [010] direction and $k_{SL}$, obtained via nextnano++ simulations. The red lines represent hole levels, and blue lines represent electron levels. The shown energy gaps ($E_{g}$) were calculated at the Γ point. Figures (**a**–**f**) show the lowest temperature (with 10 K precision) at which the energy level from the InSb interface (represented by dotted blue lines) occurs inside the energy gap.

**Figure 8:**
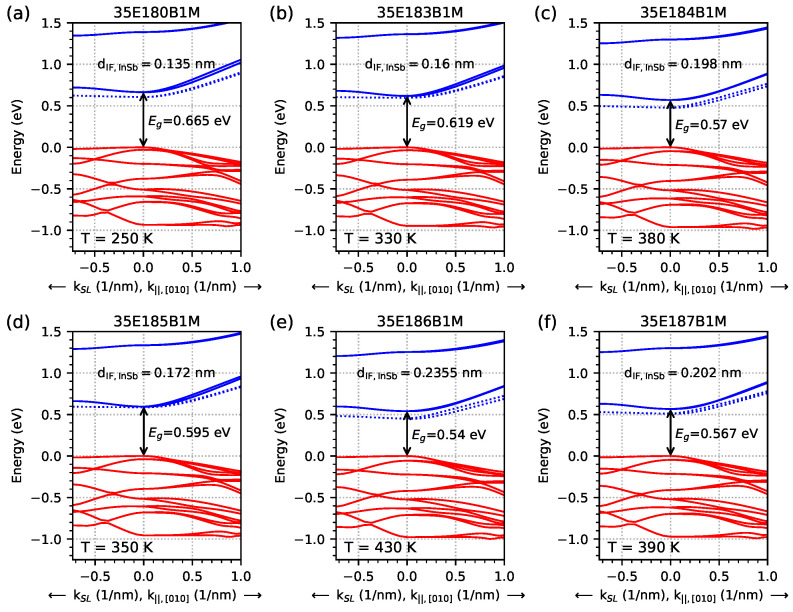
Dispersion relation for investigated samples parallel and perpendicular to the sample layers for the *k*-space [010] direction and $k_{SL}$, obtained from nextnano++ simulations. The red lines represent hole levels, and the blue lines represent electron levels. The shown energy gaps ($E_{g}$) were calculated at the Γ point. Figures (**a**–**f**) show the highest temperature (with 10 K precision) at which the energy level coming from the InSb interface (represented by dotted blue lines) occurs inside the energy gap.

**Figure 9:**
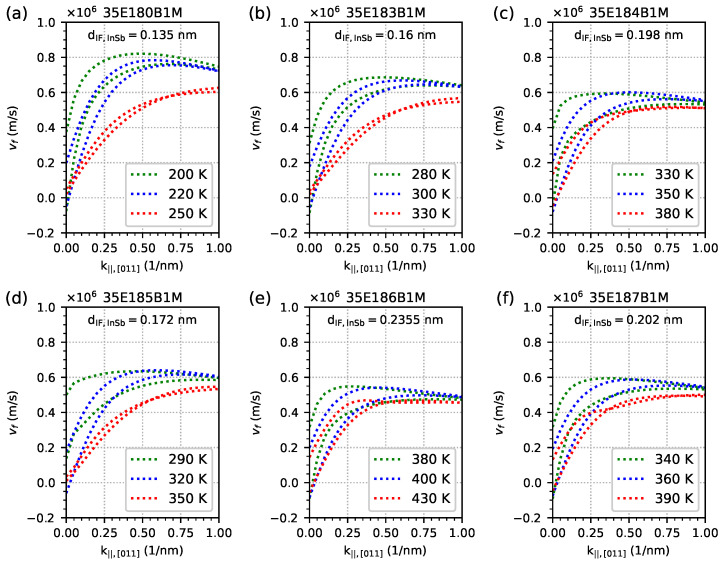
Fermi velocity $\nu_{f}$ as a function of $k_{\left\|,[010\right]}$, computed from the dispersion relation obtained from nextnano++ simulations for the investigated samples at the temperature at which the energy level coming from the InSb interface is placed inside the energy gap.

Fermi velocity $\nu_{f}$ can be obtained from the E(k) relation, $\Delta E=\hbar \nu_{f}\Delta k$ (in the linear region of the dispersion relation), as shown in Figure 7, and this type of dispersion is represented by one and the two dotted blue lines (the one with higher energy) for the $k_{\left\|[010\right]}$ direction and at the lowest temperature when EGIF occurs. Similarly to Figure 7, Figure 8 shows that the dispersion relation for the highest temperature of EGIF still exists inside the energy gap; however, its slope is smaller, and the linear dispersion is also not clearly visible. The values of the Fermi velocity $\nu_{f}$ for all structures were calculated for Δk = 0.05 nm^−1^ and plotted in Figure 9 as a function of $k_{\left\|,[010\right]}$ for three temperatures: lowest, middle, and highest temperatures at which EGIF occurs. For k=0, the calculated value of $\nu_{f}$ is around 200 km/s (or reaches negative values slightly below 0 km/s). For *k* in the range of 0–0.2…0.5 nm^−1^, the value of $\nu_{f}$ increases almost linearly to the maximum value of around 450–800 km/s, and only for higher values of *k* (within the range of 0.5–1.0 nm^−1^ does it reach a fairly constant level that is appropriate for the linear dependence of E(k)—(with a slight decrease in value for $\nu_{f}$ with respect to increased *k* values). However, when the temperature is lower (the energy values of EGIF are closer to the hole levels), the Fermi velocity $\nu_{f}$ reaches a relatively constant level for lower values of $k_{\left\|,[010\right]}$ and has a slightly higher value than for the higher temperatures (where the values of energy of EGIF are closer to the electron levels).

**Figure 10:**
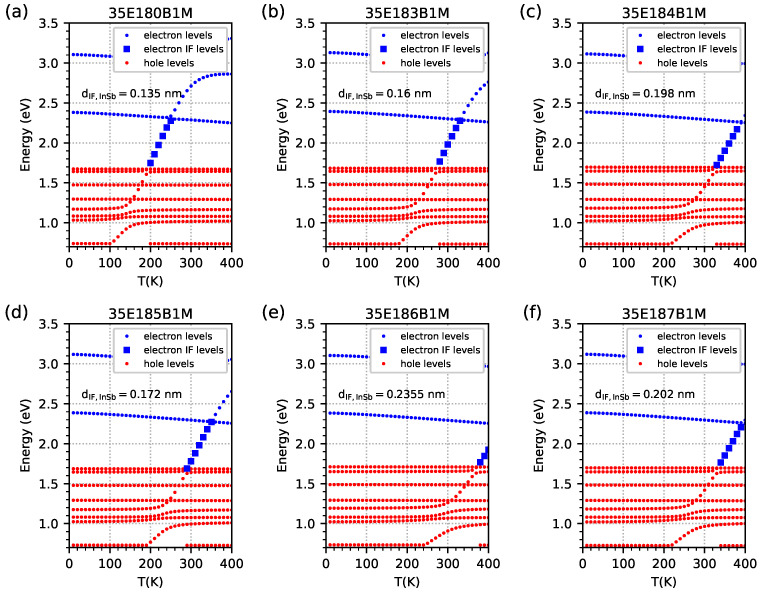
Energy spectrum obtained from nextnano++ simulations within the temperature range of 10–400 K, with 10 K intervals for investigated samples modelled as an infinite number of period structures using data from Table 1. The red dots represent energy levels for holes. The blue dots represent energy levels for electrons. The blue squares represent energy levels for electrons corresponding to the energy level located inside the energy gap for electrons coming from the InSb interface.

The computed energy spectrum at the Γ point as a function of temperature for the same investigated samples is presented in Figure 10. The EGIF and their ‘formation’ are visible in this figure. In the case of increasing temperatures, at lower temperatures, some of the hole levels (the red dots) rapidly change their energies; if the temperature is high enough, they appear inside the energy gap as a blue square, and finally, at the highest temperatures, they continue to move outside the energy gap at a ’normal’ electron level (the blue dots). The temperature range for the levels occurring inside the energy gap is the same as for the temperature range for ${\left|\Psi \right|}^{2}$ corresponding to EGIF in Figure 6. Figure 6 also shows the temperature dependence of EGIF in the IF InSb layer of each of six samples with different IF InSb widths—except samples 35E184B1M and 35E187B1M with similar IF InSb widths.

**Figure 11:**
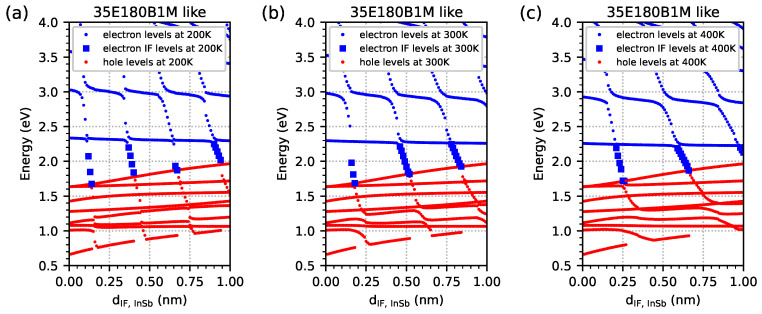
Energy spectrum obtained from nextnano++ simulations at temperatures of (**a**) 200 K, (**b**) 300 K, and (**c**) 400 K for parameters similar to the 35E180B1M sample modelled as an infinite number of period structures, with the $d_{\mathrm{IF},\mathrm{InSb}}$ width within the range of 0.01–1.00 nm at 0.01 nm intervals. The red dots represent energy levels for holes. The blue dots represent energy levels for electrons. The blue squares represent the energy levels of electrons corresponding to the energy level located inside the energy gap with respect to electrons coming from the InSb interface.

For this purpose, a series of nextnano++ simulations were performed for a sample with parameters similar to 35E180B1M, but only the IF InSb width was varied. Figure 11 shows the energy spectrum (similarly to Figure 10) versus the width of IF InSb in the range of 0.01–1.00 nm, with 0.01 nm intervals at three temperatures of (a) 200 K, (b) 300 K, and (c) 400 K for parameters that are similar to the 35E180B1M sample, modelled as an infinite number of period structures. For the temperatures of 200 K/300 K/400 K/in Figure 11, the energy level of the electrons in the energy gap occurs in 4/3/2 ‘groups’, respectively, and it is located at the larger InSb IF width at higher temperatures. For the temperature of 200 K in Figure 11a, the first group of these levels occurs at an IF InSb width within the range of 0.12–0.15 nm, the second group at 0.37–0.40 nm, and the third group at 0.66–0.67 nm (for temperatures of 300 K and 400 K, the first group is 0.16–0.18 nm and 0.21–0.27 nm, respectively)—which is consistent with the results for all investigated samples presented in Figure 6, Figure 7, and Figure 8. The groups of levels are placed (more or less) at odd multiples of the IF InSb’s width corresponding to the first group of levels (e.g., at a temperature if 200 K: 0.12 nm, 3 × 0.12 nm = 0.36 nm, and 5 × 0.12 nm = 0.60 nm).

**Figure 12:**
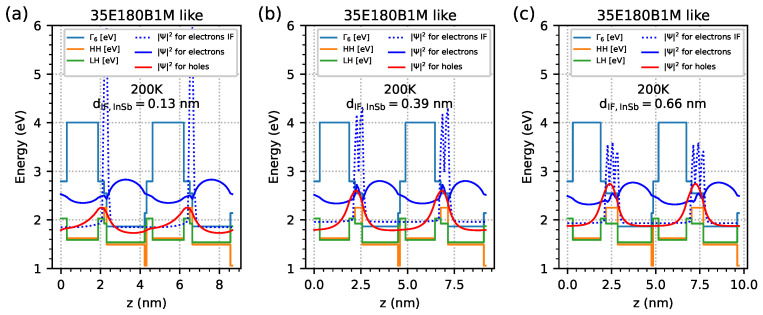
Band edge’s diagram and probability density function ${\left|\Psi \right|}^{2}$ of electrons and holes obtained at a temperature of 200 K from nextnano++ simulations for modelled samples, such as 35E180B1M, as an infinite number of period structures with a $d_{\mathrm{IF},\mathrm{InSb}}$ width: (**a**) 0.13 nm, (**b**) 0.39 nm, and (**c**) 0.66 nm. The red line represents holes ${\left|\Psi \right|}^{2}$, and the blue line represents electrons ${\left|\Psi \right|}^{2}$. The dotted blue line represents the electron ${\left|\Psi \right|}^{2}$ coming from the InSb interface.

Figure 12 shows the band edge’s diagram and the probability density function ${\left|\Psi \right|}^{2}$ of electrons and holes obtained from simulations at a temperature of 200 K for a modelled sample similar to 35E180B1M with an infinite number of period structures with different $d_{\mathrm{IF},\mathrm{InSb}}$ widths: (a) 0.13 nm, (b) 0.39 nm, and (c) 0.66 nm. The red line represents the ${\left|\Psi \right|}^{2}$ holes, and the blue line represents the ${\left|\Psi \right|}^{2}$ electrons. The dotted blue line represents electron ${\left|\Psi \right|}^{2}$ coming from EGIF, with one/two/three maxima with respect to ${\left|\Psi \right|}^{2}$ for a width $d_{\mathrm{IF},\mathrm{InSb}}$ equal to 0.13/0.39/0.66 nm respectively.

**Figure 13:**
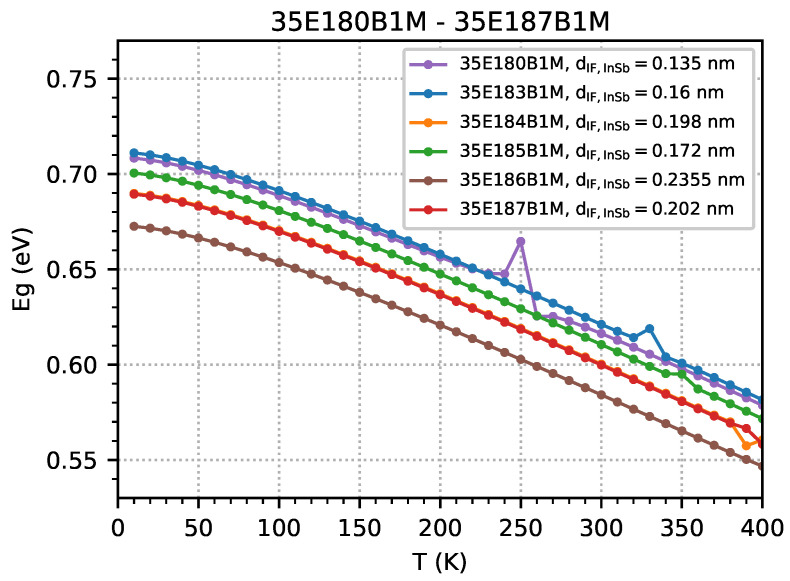
The energy gap ($E_{g}$) as a function of temperature, obtained from nextnano++ simulations over a temperature range of 10–400 K with 10 K steps for the investigated samples modelled as an infinite number of period structures using the data from Table 1. The presented values of the energy gap were obtained at the Γ point, calculated as the difference between the energy of the lowest electron level and the energy of the highest hole level (excluding the electron energy level from the InSb interface).

**Figure 14:**
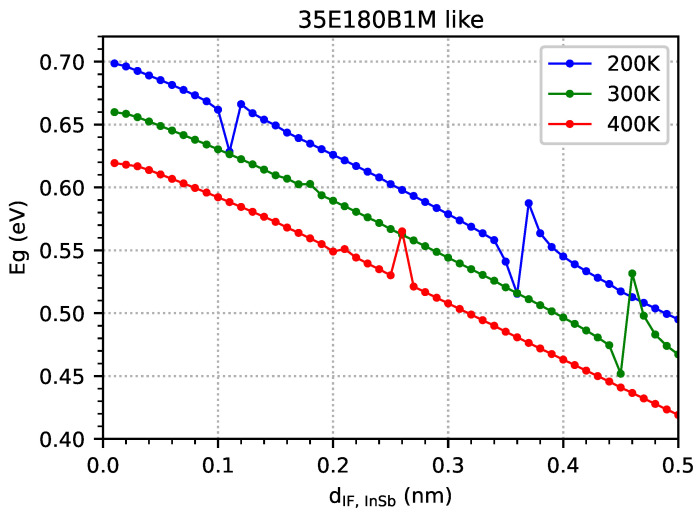
The energy gap ($E_{g}$) as a function of the InSb interface’s width in the range of 0.01–0.50 nm with 0.01 nm intervals was obtained from nextnano++ simulations at temperatures of 200 K, 300 K, and 400 K for sample parameters such as 35E180B1M, and they were modelled as an infinite number of period structures.

It is worth noting that the simulation results indicate that the energy gap decreases with an increase in temperature (Figure 13) or IF InSb width (Figure 14). However, when the temperature increases, a decrease in the electron level contributes to gap reductions (Figure 10), and when the IF InSb’s width increases, it contributes most to the increase in the hole level (Figure 11).

**Figure 15:**
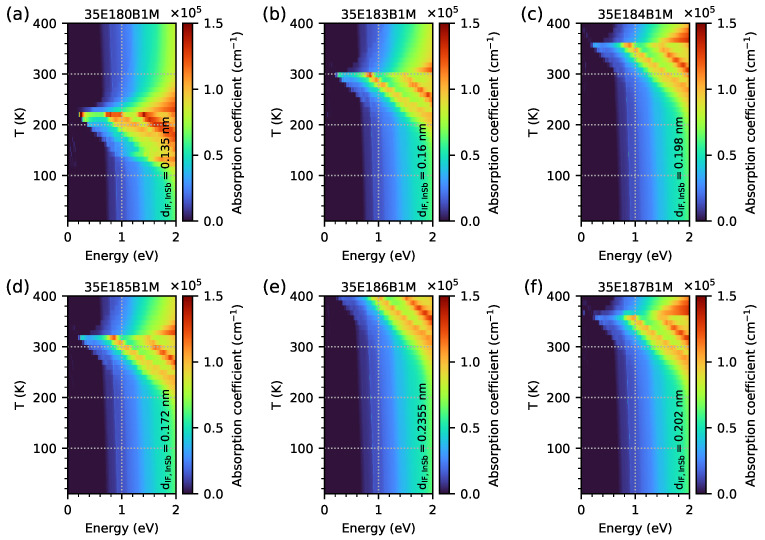
The absorption coefficient was obtained from nextnano++ simulations over a temperature range of 10–400 K, with 10 K intervals, and photon energies ranging from 0 to 2 eV. The investigated samples were modelled as a 5-period structure using the data from Table 1.

**Figure 16:**
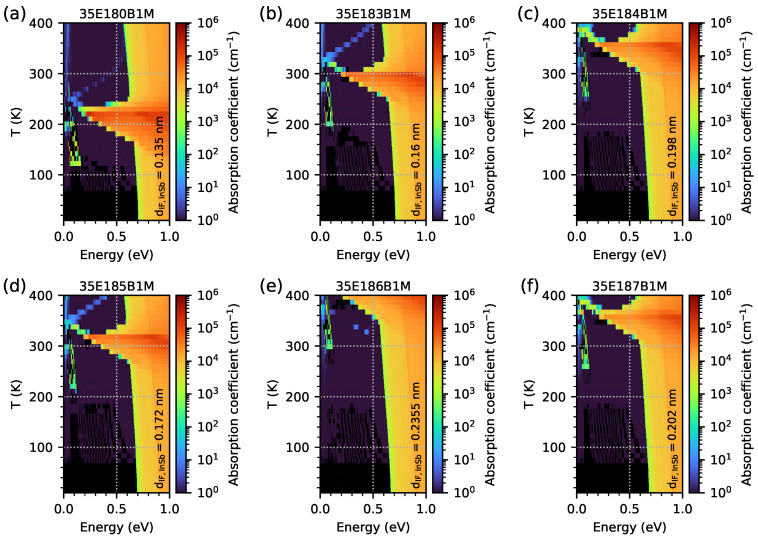
The absorption coefficient, presented on a logarithmic scale, was obtained from nextnano++ simulations over a temperature range of 10–400 K with 10 K intervals and photon energies ranging from 0 to 1 eV. The investigated samples were modelled as a 5-period structure using the data from Table 1.

In order to check the optical properties and the influence of EGIF levels on them, the optical absorption coefficient was simulated using the quantum approach for all investigated samples modelled as five-period M-structures with a 20 nm AlSb buffer and a 1 μm GaSb substrate (however, for the quantum and the optical calculation, the buffer and substrate are not included). The simulation grid was 0.1 nm for the periodic M-structures and buffer and 10 nm for the substrate. The results for the optical absorption coefficient as a function of temperature within the range of 10–400 K, with intervals of 10 K in photon energy within the range 0–2 eV for all investigated samples, are shown as a colour maps in Figure 15. Two important phenomena are visible. Firstly, the optical absorption edges are around 0.6–0.7 eV (as a vertical, slightly diagonal line separating the black and blue areas), and they are comparable to the $E_{g}$ previously calculated with dispersion relation data. Secondly, a significant increase in the absorption coefficient is visible for temperature ranges close to the temperature range where EGIF levels occur for each sample with respect to the 0–2 eV photon energy range (for sample 35E186B1M, the temperature at 400 K is too low for exhibiting a decrease in absorption at high temperatures). The increase in the absorption coefficient (especially for photon energies higher than the absorption edge) occurs at a slightly lower temperature than the temperatures assumed to be the lowest for IF, as shown in Figure 7. On the other hand, the maximum absorption for the lowest photon energy occurs at a temperature close to the middle between the lowest and highest temperatures for the EGIF shown in Figure 7 and Figure 8, respectively. Above this temperature, there is a sharp decrease in absorption, especially for photon energies that are lower than the absorption edge (and with absorption decaying relatively slowly for photon energies of >1 eV). To better visualise the absorption spectrum near the absorption edge as a function of temperature, the same data are plotted on a logarithmic scale within the photon energy range of 0–1 eV in Figure 16.

**Figure 17:**
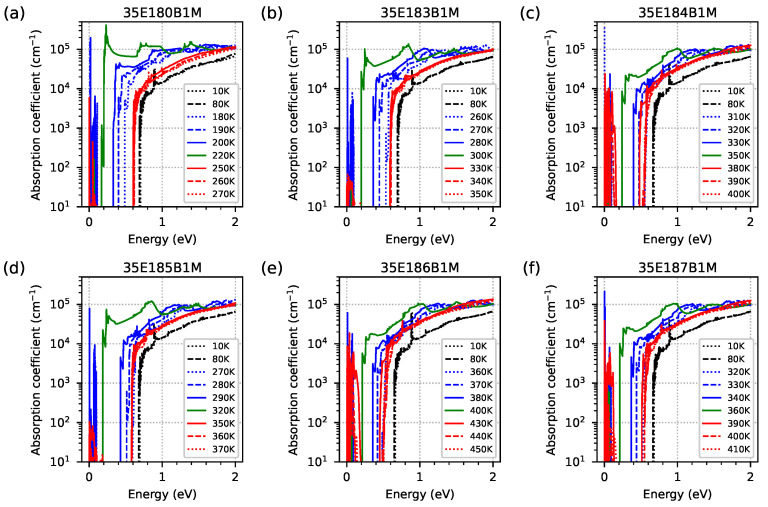
The optical absorption spectrum, presented on a logarithmic scale, was obtained from nextnano++ simulations for several temperatures within the range of 10–450 K and photon energies ranging from 0 to 2 eV; this is for the investigated samples modelled as a five-period structure using the data from Table 1. The red/green/blue solid line shows the optical absorption coefficient at the lowest/intermediate/highest temperature (with an accuracy of 10 K), where the energy level coming from the InSb interface occurs inside the energy gap.

**Figure 18:**
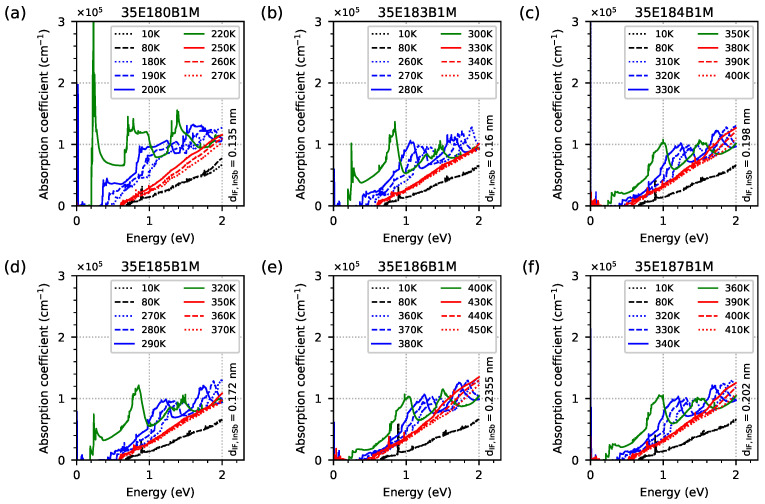
The optical absorption spectrum, obtained from nextnano++ simulations for several temperatures within the range of 10–450 K and photon energies ranging from 0 to 2 eV, for the investigated samples modelled as a 5-period structure using the data from Table 1. The red/green/blue solid line shows the optical absorption coefficient at the lowest/intermediate/highest temperature (with an accuracy of 10 K), where the energy level coming from the InSb interface occurs inside the energy gap.

Figure 17 shows, on a logarithmic scale, the optical absorption spectrum obtained for several temperatures within the range of 10–450 K for all samples investigated in order to better visualise the shape of the absorption spectra, the absorption edge for the temperatures corresponding to EGIF (the three solid lines), and the slightly lower and higher (the dashed lines) temperatures. The same data as shown in Figure 17 are presented in Figure 18 on a linear scale where a significant increase in the optical absorption coefficient is shown for the lowest temperatures when EGIF exists (presented in Figure 7) and when temperatures are close to the middle values, between the lowest and highest temperatures (presented in Figure 8). The absorption spectra for the highest temperatures when EGIF still exists are comparable to the spectra obtained at higher temperatures.

**Figure 19:**
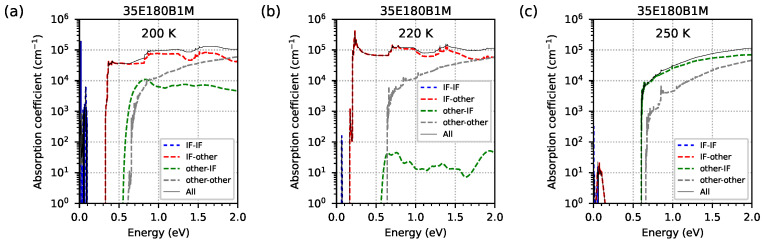
The optical absorption spectrum, presented on a logarithmic scale, was obtained from nextnano++ simulations for temperatures of the case in which electron energy levels from the InSb interface occur inside the energy gap: (**a**) 200 K, (**b**) 220 K, and (**c**) 250 K. This is for the investigated sample 35E180B1M, modelled as a 5-period structure using the data from Table 1. The dashed lines show the contribution to the optical absorption coefficient, distinguishing whether the initial (final) levels come from (or do not come from) the InSb interface. The solid black line shows the total optical absorption coefficient as the sum of the individual contributions represented by the dashed lines.

**Figure 20:**
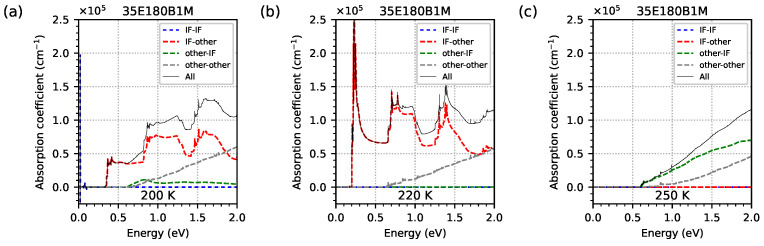
The optical absorption spectrum, obtained from nextnano++ simulations for temperatures when electron energy levels from the InSb interface occur inside the energy gap: (**a**) 200 K, (**b**) 220 K, and (**c**) 250 K. This is for the investigated sample 35E180B1M, modelled as a 5-period structure using the data from Table 1. The dashed lines show the contribution to the optical absorption coefficient, distinguishing whether the initial (final) levels come from (or do not come from) the InSb interface. The solid black line shows the total optical absorption coefficient as the sum of the individual contributions represented by dashed lines.

The contribution of optical transitions involving EGIF levels in the absorption spectrum was analysed. Figure 19 shows, relative to a logarithmic scale (and Figure 20 relative to linear scales), the optical absorption spectrum obtained from nextnano++ simulations for temperatures of (a) 200 K, (b) 220 K, and (c) 250 K for the investigated sample 35E180B1M. The solid black line (denoted as ‘All’) shows the total optical absorption coefficient as a sum of the individual contributions shown in dashed lines. For the lower and middle temperatures of (a) 200 K and (b) 220 K, the main contributions come from transitions from EGIF to other levels (the red dashed line denoted as ‘IF-other’). However, for the highest temperature at which EGIF is still present ((c) 250 K), the main contribution comes from transitions from other levels to EGIF (the green dashed line denoted as ‘other-IF’), and contributions from EGIF to other levels (the red dashed line) are completely damped for photon energies greater than about 0.15 eV (for photon energies < 0.15 eV, relatively low absorption occurs; see Figure 19). The contributions of intraband transitions (the blue dashed line denoted as ‘IF-IF’) for photon energy within the range of 0.1–2.0 eV are absent at these three temperatures (only visible as sharp absorption peaks for photon energy < 0.1 eV, with the highest level at a temperature of 200 K and the lowest level at a temperature of 250 K; see Figure 19). The contribution of transitions between non-EGIF levels (the grey dashed line denoted as ‘other-other’) is approximately at the same level for all these three temperatures.

**Figure 21:**
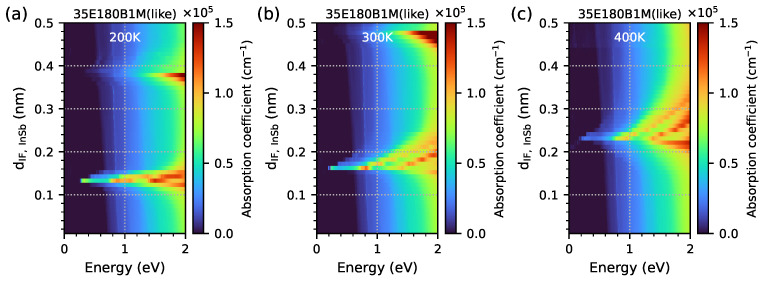
The optical absorption coefficient, obtained from nextnano++ simulations at temperatures of (**a**) 200 K, (**b**) 300 K, and (**c**) 400 K, for parameters such as the 35E180B1M sample modelled as a 5-period structure, with the $d_{\mathrm{IF},\mathrm{InSb}}$ width ranging from 0.01 to 0.50 nm at 0.01 nm intervals and photon energies ranging from 0 to 2 eV.

In order to check the influence of the IF InSb’s width on the absorption spectrum, a series of simulations for five-period M-structures with 80 electron and hole levels (and a 33 × 33 point grid in *k*-space) was performed for a sample with parameters similar to 35E180B1M, but only the IF InSb’s width was varied within the range 0.01–0.50 nm, with 0.01 nm intervals at three temperatures. The selected range of the InSb interface’s thickness is based on actual measurements of this thickness using the HRXRD method. In reality, 0.5 nm corresponds to an interfacial thickness of approximately 1.5 monolayer (ML). Such thick interfaces are not actually used, which is why this is the upper limit considered in the calculations presented. As can be observed in Table 1, the actual interface’s thicknesses are at a level of less than one atomic monolayer, i.e., a thickness of about 0.3 nm. The determined thicknesses are accurate relative to 0.01 nm; hence, this value was also adopted in calculations as the smallest step. Figure 21 shows the results obtained for the following temperatures: (a) 200 K, (b) 300 K, and (c) 400 K. Similarly to Figure 15 and Figure 16, the optical absorption edges are clearly visible. The first maximum of the optical absorption at temperatures of 200/300/400 K exists at 0.14/0.16/0.24 nm of $d_{\mathrm{IF},\mathrm{InSb}}$—which is consistent with the data presented in Figure 11. The second maximum of the optical absorption at a temperature of 200 K is also visible (but weaker) for IF InSb widths of approximately 0.38 nm, and this value is also comparable with the data presented in Figure 11 (at a temperature of 300 K, higher absorption is visible for IF InSb widths within the range of 0.45–0.48 nm).

## 4. Conclusions

In this paper, we investigated specially designed InAs/GaSb/AlSb/GaSb M-structures modelled as T2SL structures, and they were proposed as active materials in IR detectors for the SWIR region. We calculated dispersion relations, energy gaps, Fermi velocities, probability density functions ${\left|\Psi \right|}^{2}$, and absorption coefficients for all six investigated M-structures using nextnano++ software over a wide temperature range from 10 K to 400 K with 10 K steps (or for several temperatures). In addition, we performed calculations of the absorption coefficient and energy spectrum for one M-structure (35E180B1M) with varying InSb-like interface widths over a wide range (up to 0.5 nm with 0.01 nm steps) for the three temperatures. Real material samples of the investigated M-structures were grown using MBE technology, and HRXRD measurements were performed: 004 reflection 2Θ−ω scans for all samples and RSM maps for the two investigated samples (35E183B1M and 35E185B1M). The results of the HRXRD measurements indicated the thickness values of each layer and confirmed the very good quality of the crystallographic properties of the grown M-structure material samples.

Detailed conclusions from the results obtained from the nextnano++ simulations of the investigated samples indicate the following:

1. EGIF levels form within a specific temperature range and a specific range of IF InSb widths. The specific width of IF InSb for EGIF is higher at higher temperatures. The specific temperature for EGIF is higher for higher IF InSb widths.
2. In simulations with an infinite number of M-structure periods, the number of EGIF levels is equal to two. In simulations with a five-period M-structure, the number of EGIF levels is equal to 10. The results of the dispersion relation and the EGIF are comparable between these two types of simulations.
3. The values of the calculated Fermi velocity $\nu_{f}$ for the EGIF levels for the $k_{\left\|[010\right]}$ direction are as follows: for k=0, they range from slightly below 0 to about 500 km/s (average about 200 km/s) depending on the temperature and M-structure parameters. For $k_{\left\|[010\right]}$ in the range of 0–0.2…0.5 nm^−1^, it increases almost linearly for a maximum value of approximately 450…800 km/s (for lower temperatures, the maximum value of $\nu_{f}$ is higher and is achieved for smaller values of $k_{\left\|[010\right]}$). For the value of $k_{\left\|[010\right]}$ in the range of 0.2–1.0 nm^−1^, velocity $\nu_{f}$ reaches a relatively constant level corresponding to linear dependence E(k)—with a slight decrease in the value of $\nu_{f}$ with an increase in the value of $k_{\left\|[010\right]}$.
4. The EGIF manifests as the maxima of ${\left|\Psi \right|}^{2}$ for electrons located in the IF InSb layer. At specific temperatures, EGIF exists for an odd multiplication of the smallest IF InSb base’s width when the first EGIF occurs at this temperature. For the ‘first’ EGIF, there is only one maximum for the EGIF ${\left|\Psi \right|}^{2}$ function. For the ‘second’ EGIF, there are two maxima, and for the ‘third’ EGIF, there are three maxima. The amplitudes of the ‘second’/’third’ maxima are approximately two/three times lower than the ‘first’ maxima.
5. The computed optical absorption coefficient indicates the optical absorption edge corresponding to the computed $E_{g}$. The maxima of absorption versus temperature at specific widths of the IF InSb correspond to the computed EGIF—a high increase in optical absorption at the lowest temperature (and also at lower temperatures) and the middle temperature where EGIF is present. The increase in optical absorption within this temperature range is mainly due to the transitions from EGIF to other electron levels (at the highest temperature where EGIF still exists, the main contribution to optical absorption is from transitions from hole levels to the EGIF).
6. The absorption maxima as a function of the IF InSb’s width at specific temperatures correspond to the calculated EGIF. At temperatures of 200 K and 300 K, two optical absorption maxima occur for the IF InSb width within the range of 0.01–0.50 nm.

## Figures and Tables

**Table 1:** The results of the simulation of the HRXRD measurements of the M-structures InAs/GaSb/AlSb/GaSb synthesized by MBE technology with respect to the thickness of monolayers (MLs) and both the InSb-like and GaAs-like IFs of each component and the lattice mismatch of the M-structures versus the GaSb substrate.

Sample	InAs	InSb-IF	GaSb	AlSb	GaSb	GaAs-IF	Δa/a
No.	(ML)	(ML)	(ML)	(ML)	(ML)	(ML)	10^−3^
35E180B1M	6.40	0.45	1	5.27	1	0.33	−0.93
35E183B1M	6.14	0.53	1	5.33	1	0.33	−0.13
35E184B1M	6.18	0.66	1	5.19	1	0.33	0.83
35E185B1M	6.21	0.57	1	5.22	1	0.33	0.13
35E186B1M	6.13	0.79	1	5.08	1	0.33	1.80
35E187B1M	6.13	0.67	1	5.14	1	0.33	0.93

## Data Availability

The data that support the findings of this study are available from the corresponding author upon reasonable request (The research was carried out as part of the project and is the author’s data).
